# Supplementary material for: Sandalwood Sesquiterpene (Z)-α-Santalol Exhibits In Vivo Efficacy Against Madurella mycetomatis in Galleria mellonella Larvae
Source: Molecules. 2025 Oct 15;30(20):4090. doi: 10.3390/molecules30204090 (PMC12566311; doi:10.3390/molecules30204090)
Supplement: Supplementary file 1 [file molecules-30-04090-s001.zip › molecules-3819055-supplementary.pdf]

Supplementary Materials

Sandalwood Sesquiterpene (Z)-*α*-Santalol Exhibits In Vivo Efficacy Against *Madurella mycetomatis* in *Galleria mellonella* Larvae

Shereen O. Abd Algaffar [1], Stephan Seegers [2], Shaoqin Zhou [3], Prabodh Satyal [4], William N. Setzer [5], Thomas J. Schmidt [2,*] and Sami A. Khalid [1,*]

[1] Faculty of Pharmacy, University of Science and Technology, Omdurman 14415, Sudan; phd_sh086@hotmail.com

[2] University of Münster, Institute of Pharmaceutical Biology and Phytochemistry (IPBP), PharmaCampus, Corrensstrasse 48, D-48149 Münster, Germany; s_seeg03@uni-muenster.de

[3] Radboudumc—CWZ Centre of Expertise for Mycology, 6525 Nijmegen, The Netherlands; shaoqin.zhou@radboudumc.nl

[4] Aromatic Plant Research Center, Lehi, UT 84043, USA; psatyal@aromaticplant.org

[5] Department of Chemistry, University of Alabama in Huntsville, Huntsville, AL 35899, USA; wsetzer@chemistry.uah.edu

* Correspondence: thomschm@uni-muenster.de (T.J.S.); khalidseek@hotmail.com (S.A.K.)

Figure S1. EI-QTOF mass spectrum of (+)-(*Z*)-*α*-santalol (70eV)

Figure S2. ^{1}H-NMR-spectrum of (+)-(*Z*)-*α*-santalol ($CDCl_3$, 600 MHz)

Figure S3. ^{13}C-NMR-spectrum and chemical structure of (+)-(*Z*)-*α*-santalol ($CDCl_3$, 150 MHz)

Figure S4. EI-QTOF mass spectum of (-)-(Z)-β-santalol (70eV)

Figure S5. ^{1}H-NMR-spectrum of (-)-(Z)-β-santalol ($CDCl_3$, 600 MHz)

Figure S6. ^{13}C-NMR-spectrum and chemical structure of (-)-(Z)-β-santalol ($CDCl_3$, 150 MHz)

Table S1: EI-QTOF MS data of (+)-(Z)-α-santalol and (-)-(Z)-β-santalol

Table S2: ^{1}H- and ^{13}C-NMR data of (+)-(Z)-α-santalol and (-)-(Z)-β-santalol

Table S3. Death records of *Galleria mellonella* larvae infected with *Madurella mycetomatis*, and treated with Royal Hawaiian sandalwood oil, *α*-santalol, β-santalol and itraconazole

Table S4. Death records of *Galleria mellonella* larvae injected with Royal Hawaiian sandalwood oil, *α*-santalol, β-santalol and itraconazole

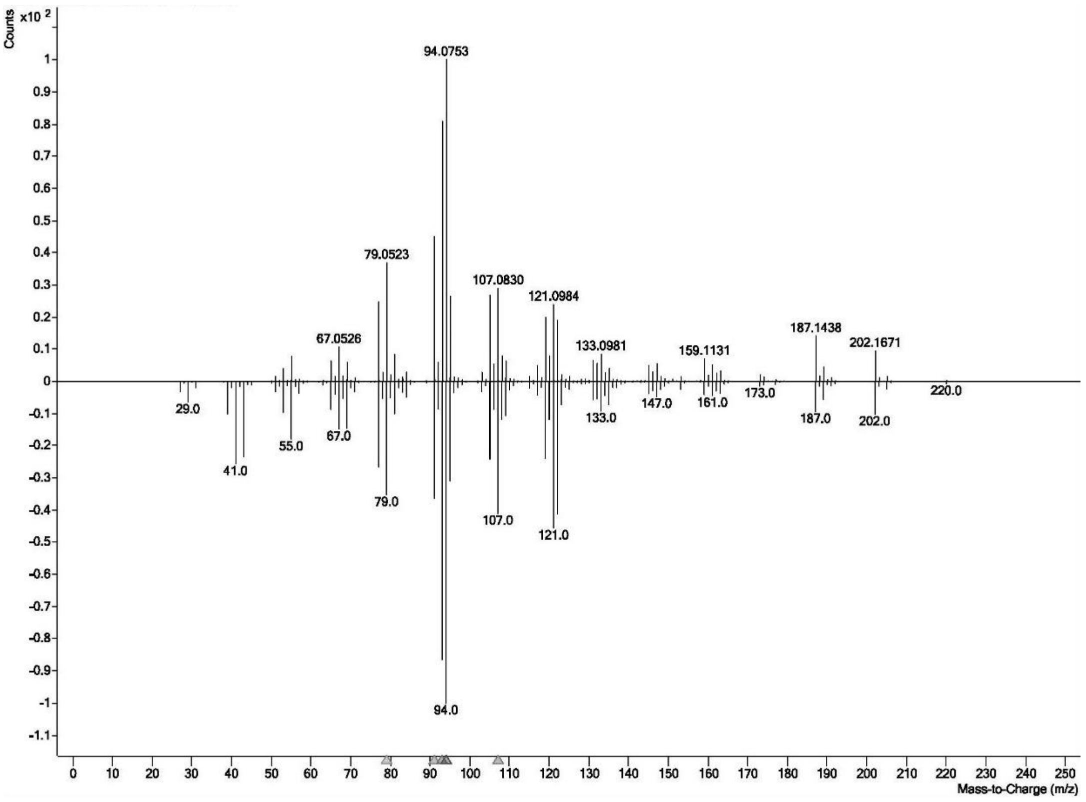

Figure S1. EI-QTOF mass spectrum of (+)-(*Z*)-α-santalol (70eV)

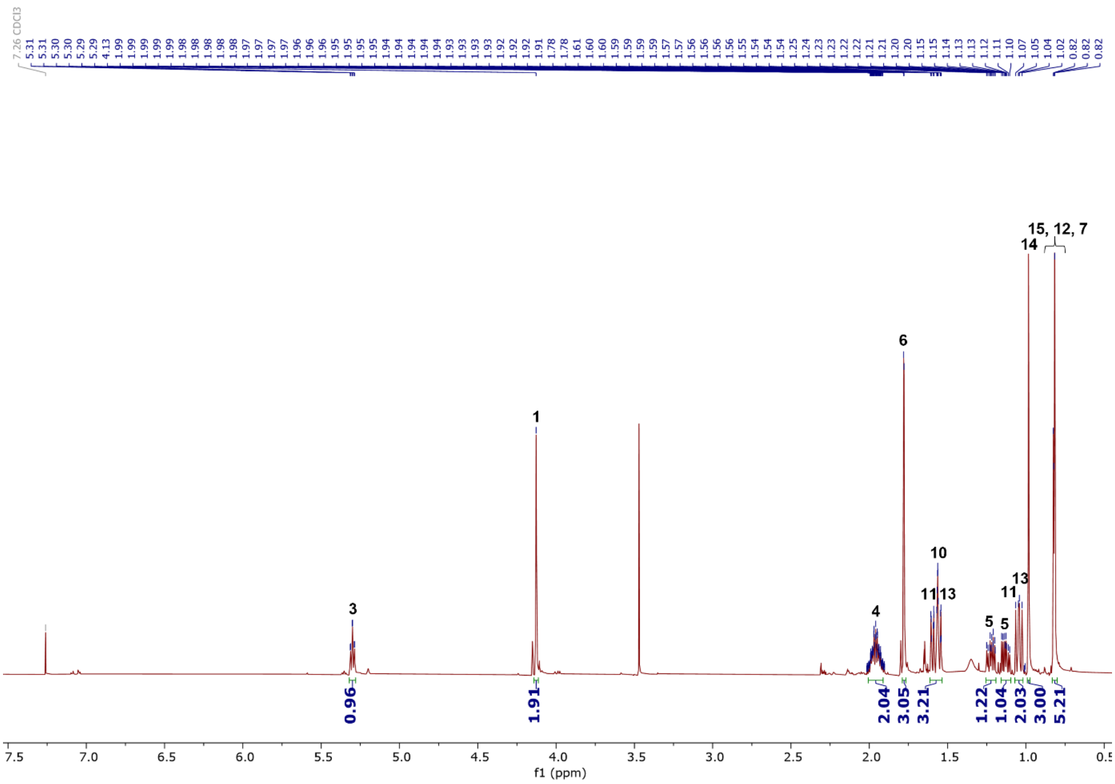

Figure S2. ^{1}H-NMR-spectrum of (+)-(*Z*)-α-santalol ($CDCl_3$, 600 MHz)

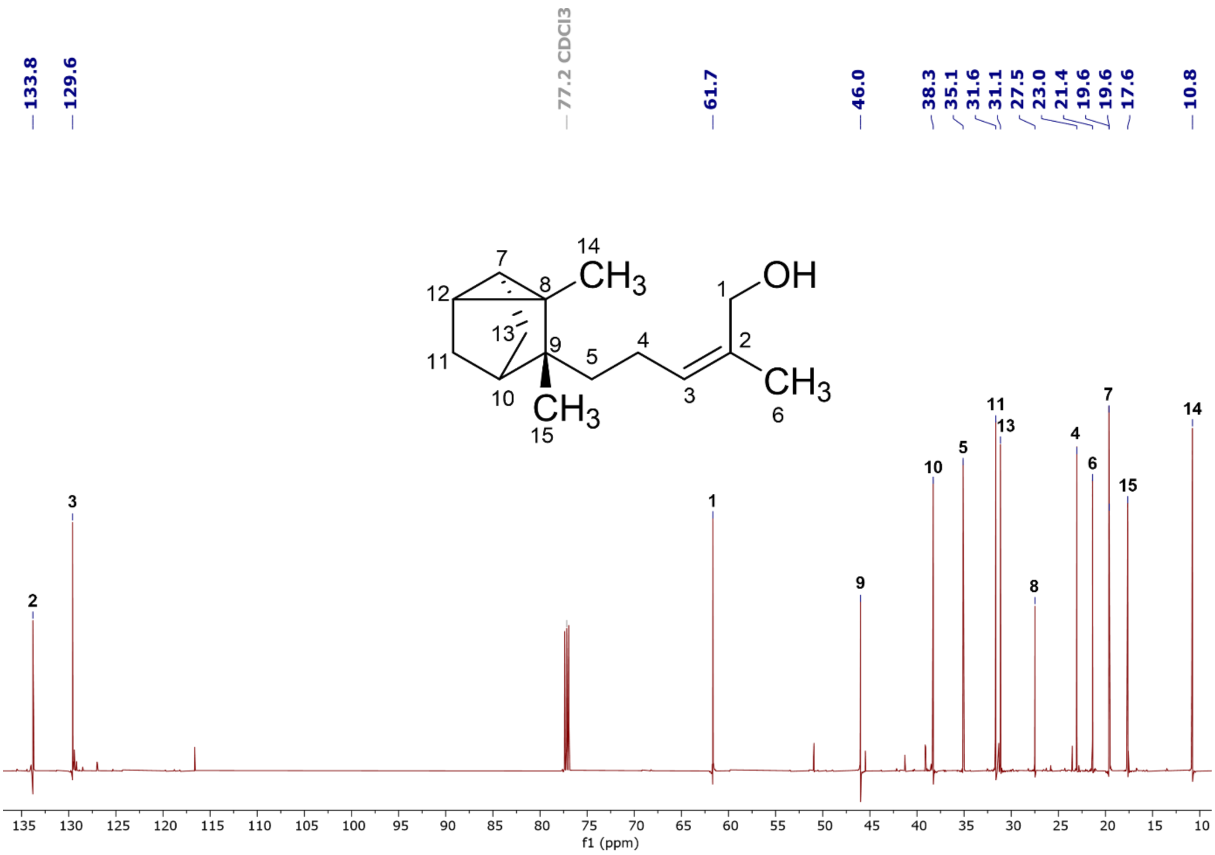

Figure S3. ^{13}C-NMR-spectrum and chemical structure of (+)-(*Z*)-*α*-santalol ($CDCl_3$, 150 MHz)

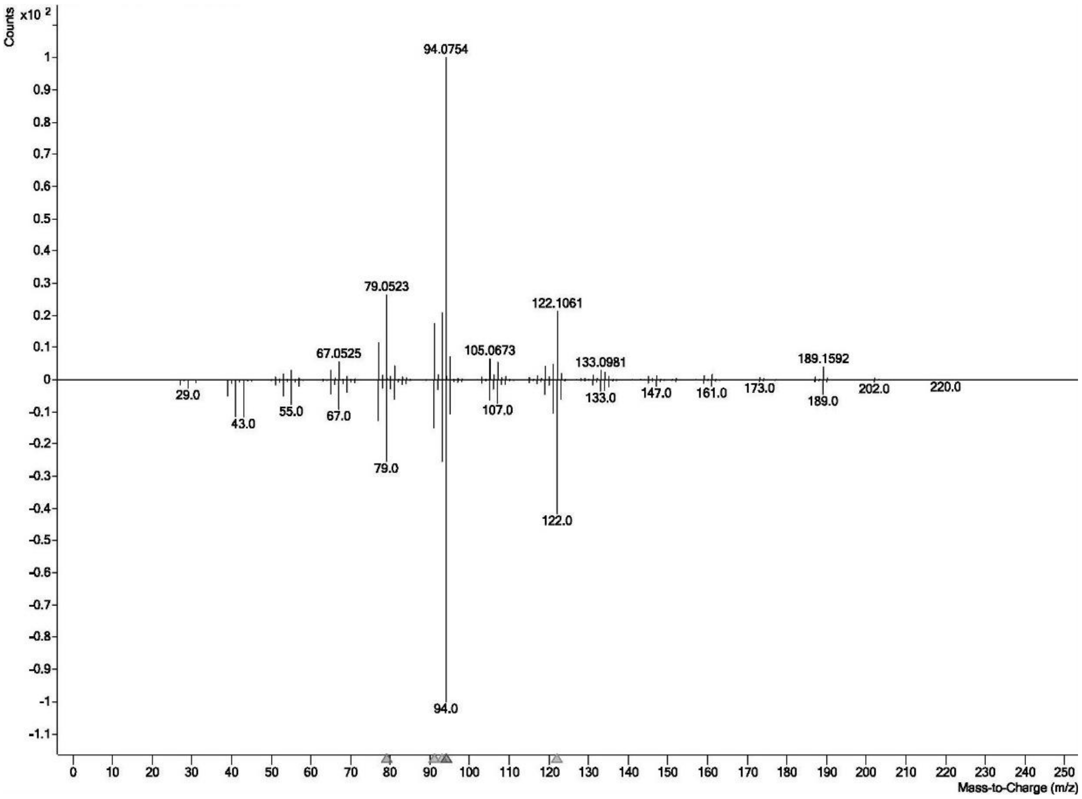

Figure S4. EI-QTOF mass spectum of (-)-(*Z*)-β-santalol (70eV)

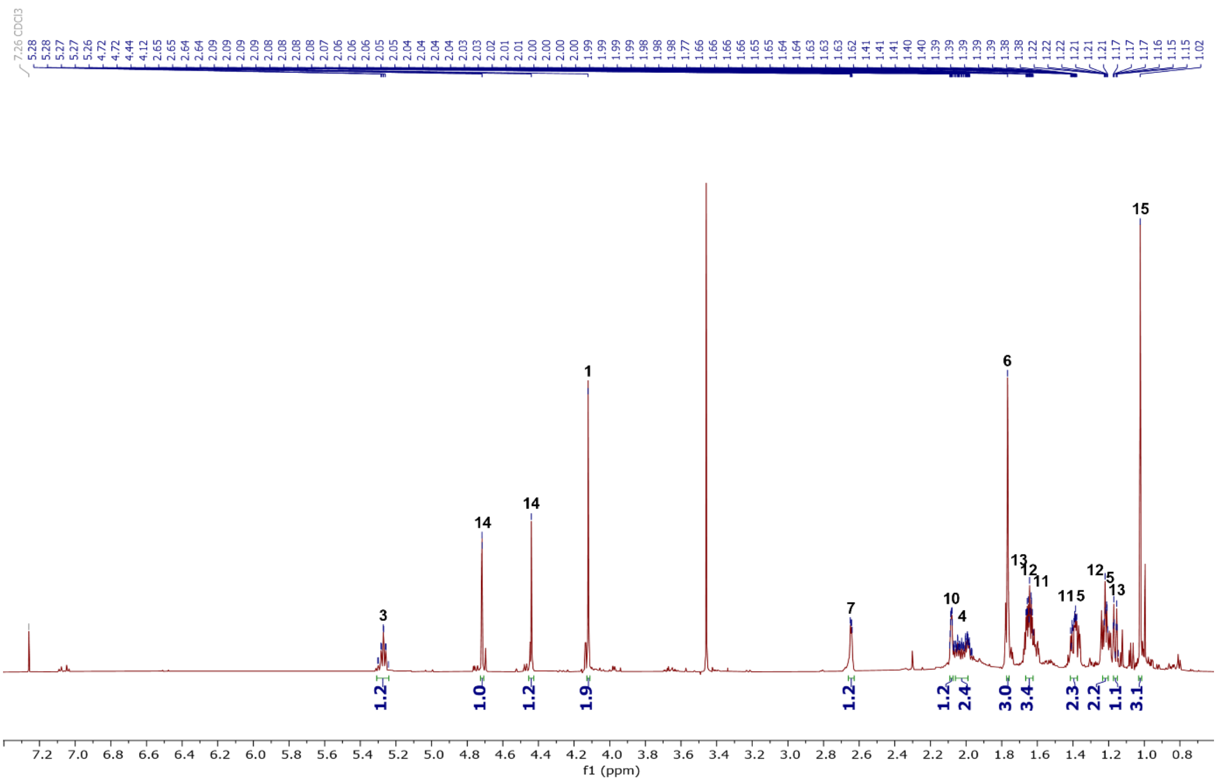

Figure S5. ^{1}H-NMR-spectrum of (-)-(*Z*)-β-santalol ($CDCl_3$, 600 MHz)

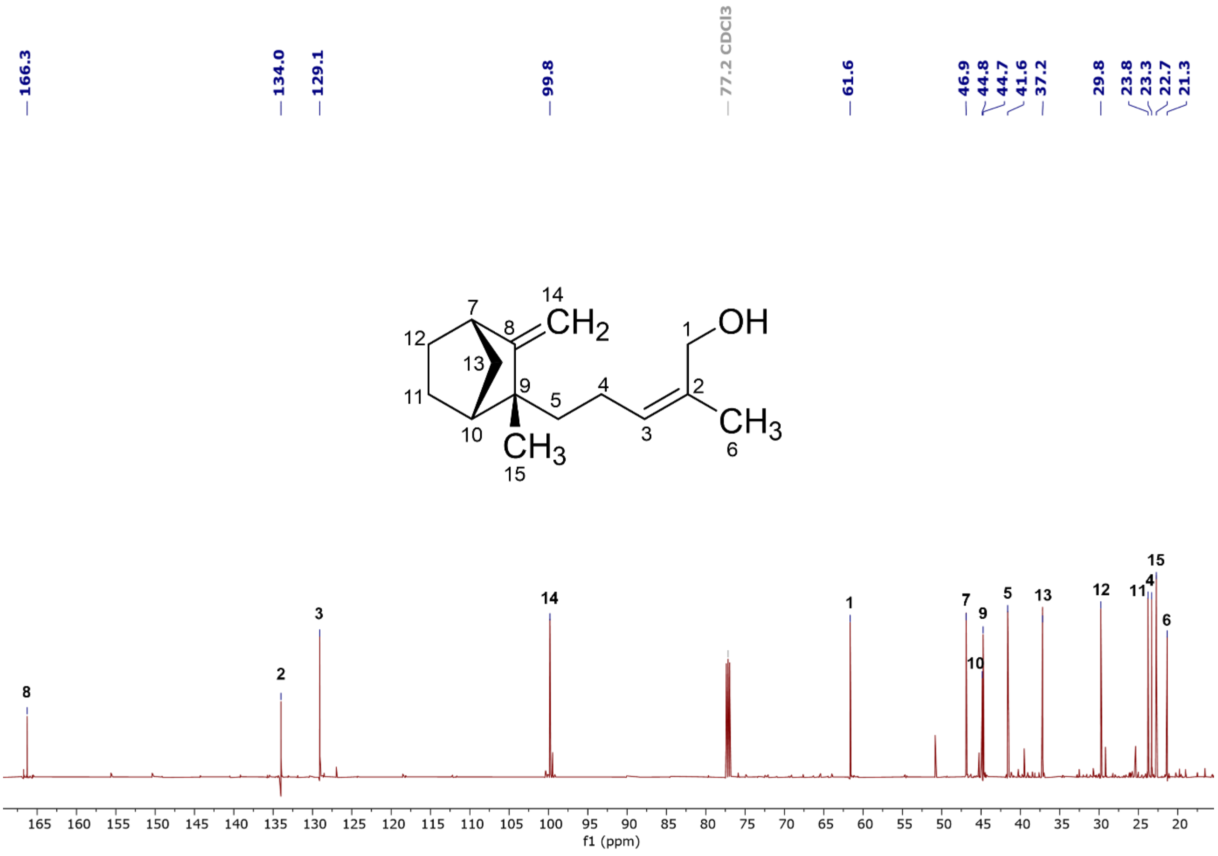

Figure S6. ^{13}C-NMR-spectrum and chemical structure of (-)-(*Z*)-β-santalol ($CDCl_3$, 150 MHz)

Table S1. EI-QTOF MS data of (+)-(Z)-α-santalol and (-)-(Z)-β-santalol

**(+)-(*Z*)-α-santalol**		**(-)-(*Z*)-β-santalol**	
**m/z**	**abundance**	**m/z**	**abundance**
77.0367	34.3	67.0527	6.2
79.0523	52.0	77.0368	13.9
91.0521	65.2	79.0524	30.6
93.0681	92.1	91.0521	21.9
94.0759	100	93.0678	28.6
95.0828	35.8	94.0755	100
105.0674	36.8	95.0794	6.9
107.083	40.4	105.0674	8.9
119.0827	27.0	121.0984	6.1
121.0983	32.6	122.1062	20.5

Table S2. ^{1}H- and ^{13}C-NMR data of (+)-(Z)-α-santalol and (-)-(Z)-β-santalol

	**(+)-(*Z*)-α-santalol**		**(-)-(*Z*)-β-santalol**	
**Position**	**δC (ppm)**	**δH (ppm)**	**δC (ppm)**	**δH (ppm)**
1	61.7	4.13	61.6	4.12
2	133.8	-	134.0	-
3	129.6	5.30	129.1	5.27
4	23.0	1.96	23.3	2.05<br>1.99
5	35.1	1.22<br>1.13	41.6	1.39<br>1.21
6	21.4	1.78	21.3	1.77
7	19.6	0.82	46.9	2.65
8	27.5	-	166.3	-
9	46.0	-	44.8	-
10	38.3	1.57	44.7	2.08
11	31.6	1.60<br>1.06	23.8	1.61<br>1.39
12	19.6	0.82	29.8	1.65<br>1.22
13	31.1	1.55<br>1.03	37.2	1.65<br>1.16
14	10.8	0.98	99.8	4.72<br>4.44
15	17.6	0.82	22.7	1.02

Table S3. Death records of *Galleria mellonella* larvae infected with *Madurella mycetomatis*, and treated with Royal Hawaiian sandalwood essential oil, α-santalol, β-santalol and itraconazole (records of three biological replicates ± sd). No. of larvae per group = 45. Each dead larva counted as 1. Data used to construct survival graphs in Figure 2 of main manuscript.

Group / Day	**PBS (Solvent control)**	**Infected (PBS-Growth control)**	**ITZ 5.71 μg/kg**	**RHS 64 μg/mL**	**RHS 16 μg/mL**	**RHS 4 μg/mL**	**α-San 110 μg/mL**	**α-San 55 μg/mL**	**α-San 13.75 μg/mL**	**β-San 110 μg/mL**	**β-San 55 μg/mL**	**β-San 13.75 μg/mL**
1.	2±0.57	3±1.00	15±3.60	13±3.21	14±4.04	5±1.15	4±0.57	14±1.52	13±2.08	17±2.08	13±1.52	22±2.08
2.	3±1.00	15±1.73	5±1.53	7±1.53	5±1.15	6±1.00	6±2.00	9±1.00	7±1.52	9±1.00	8±2.08	4±1.52
3.	1±0.57	5±0.57	3±1.00	2±0.57	1±.057	1±0.57	0±0.00	1±0.57	1±0.57	3±1.73	3±1.00	1±0.57
4.	2±0.57	3±1.00	2±0.57	1±0.57	0±0.00	3±1.00	0±0.00	2±0.57	2±0.57	5±0.57	1±0.57	0±0.00
5.	1±0.57	0±0.00	0±0.00	1±0.57	0±0.00	3±1.73	3±1.00	1±0.57	1±0.57	0±0.00	1±0.57	1±0.57
6.	0±0.00	1±0.57	0±0.00	0±0.00	0±0.00	2±0.57	1±0.57	3±1.00	0±0.00	2±1.15	1±0.57	2±0.57
7.	0±0.00	0±0.00	4±0.57	3±0.00	1±057	0±0.00	1±0.57	2±0.57	0±0.00	3±0.00	0±0.00	0±0.00
8.	0±0.00	2±0.57	0±0.00	2±0.57	2±1.15	1±0.57	4±0.57	3±1.00	2±1.15	1±0.57	2±0.57	2±0.57
9.	0±0.00	0±0.00	0±0.00	0±0.00	0±0.00	0±0.00	1±0.57	1±0.57	2±0.57	0±0.00	1±0.57	0±0.00
10.	0±0.00	3±1.00	1±0.57	1±0.57	1±0.57	0±0.00	1±0.57	1±0.57	2±0.57	0±0.00	2±1.15	0±0.00

RHS = Royal Hawaiian Sandalwood oil; α-San: α-santalol; β-San: β-santalol; ITZ: itraconazole.

Table S4. Death records of *Galleria mellonella* larvae injected with Royal Hawaiian sandalwood essential oil, α-santalol, β-santalol and itraconazole (records of three biological replicates ± sd). No. of larvae per group = 45. Each dead larva counted as 1. Data used to construct survival graphs in Figure 2 of main manuscript.

Group / Day	PBS (Solvent control)	RHS 64 µg/mL	RHS 16 µg/mL	RHS 4 µg/mL	α-San 110 µg/mL	α-San 55 µg/mL	α-San 13.75 µg/mL	β-San 110 µg/mL	β-San 55 µg/mL	β-San 13.75 µg/mL
1.	3±1.00	0±0.00	2±1.15	0±0.00	0±0.00	0±0.00	0±0.00	0±0.00	1±0.57	0±0.00
2.	1±0.57	0±0.00	3±1.00	2±0.57	0±0.00	7±1.53	7±2.31	4±1.52	3±1.73	0±0.00
3.	0±0.00	2±0.57	0±0.00	0±0.00	2±1.15	2±0.57	0±0.00	4±0.57	0±0.00	0±0.00
4.	0±0.00	0±0.00	0±0.00	0±0.00	0±0.00	0±0.00	0±0.00	2±0.57	0±0.00	0±0.00
5.	2±0.57	4±1.52	0±0.00	3±1.00	0±0.00	0±0.00	0±0.00	0±0.00	1±0.57	3±1.00
6.	0±0.00	0±0.00	0±0.00	0±0.00	0±0.00	1±0.57	2±1.15	2±1.15	3±1.00	2±0.57
7.	0±0.00	2±0.57	6±1.73	4±0.57	6±1.00	2±1.15	1±0.57	3±1.00	3±1.00	1±0.57
8.	0±0.00	6±1.00	3±1.73	0±0.00	-	-	-	-	-	-
9.	2±0.57	0±0.00	3±1.00	2±1.15	-	-	-	-	-	-
10.	1±0.57	0±0.00	0±0.00	0±0.00	-	-	-	-	-	-

RHS = Royal Hawaiian Sandalwood oil; α-San: α-santalol; β-San: β-santalol; ITZ: itraconazole.
